# Identification of pneumonia and influenza deaths using the death certificate pipeline

**DOI:** 10.1186/1472-6947-12-37

**Published:** 2012-05-08

**Authors:** Kailah Davis, Catherine Staes, Jeff Duncan, Sean Igo, Julio C Facelli

**Affiliations:** 1 Department of Biomedical Informatics, University of Utah, Salt Lake City, Utah, USA; 2 Utah Department of Health, Salt Lake City, Utah, USA; 3 Center for High Performance Computing, University of Utah, Salt Lake City, Utah, USA

**Keywords:** Public health informatics, Natural language processing, Surveillance, Pneumonia and influenza

## Abstract

**Background:**

Death records are a rich source of data, which can be used to assist with public surveillance and/or decision support. However, to use this type of data for such purposes it has to be transformed into a coded format to make it computable. Because the cause of death in the certificates is reported as free text, encoding the data is currently the single largest barrier of using death certificates for surveillance. Therefore, the purpose of this study was to demonstrate the feasibility of using a pipeline, composed of a detection rule and a natural language processor, for the real time encoding of death certificates using the identification of pneumonia and influenza cases as an example and demonstrating that its accuracy is comparable to existing methods.

**Results:**

A Death Certificates Pipeline (DCP) was developed to automatically code death certificates and identify pneumonia and influenza cases. The pipeline used MetaMap to code death certificates from the Utah Department of Health for the year 2008. The output of MetaMap was then accessed by detection rules which flagged pneumonia and influenza cases based on the Centers of Disease and Control and Prevention (CDC) case definition. The output from the DCP was compared with the current method used by the CDC and with a keyword search. Recall, precision, positive predictive value and F-measure with respect to the CDC method were calculated for the two other methods considered here. The two different techniques compared here with the CDC method showed the following recall/ precision results: DCP: 0.998/0.98 and keyword searching: 0.96/0.96. The F-measure were 0.99 and 0.96 respectively (DCP and keyword searching). Both the keyword and the DCP can run in interactive form with modest computer resources, but DCP showed superior performance.

**Conclusion:**

The pipeline proposed here for coding death certificates and the detection of cases is feasible and can be extended to other conditions. This method provides an alternative that allows for coding free-text death certificates in real time that may increase its utilization not only in the public health domain but also for biomedical researchers and developers.

**Trial Registration:**

This study did not involved any clinical trials.

## Background

The ongoing monitoring of mortality is crucial to detect and estimate the magnitude of deaths during epidemics, emergence of new diseases (for example, seasonal or pandemic influenza, AIDS, SARS), and the impact of extreme environmental conditions on a population such as heat waves or other relevant public health events or threats [1,2]. The surveillance of vital statistics is not a novel idea; mortality surveillance has played an integral part in public health since the London Bills of Mortality were devised in the seventeenth century [3]. The Bills served as an early warning tool against bubonic plague by monitoring deaths from the 1635 to the 1830s. Today, mortality surveillance continues to be a critical activity for public health agencies throughout the world [4-7].

Pneumonia and influenza are serious public health threats and are a cause of substantial morbidity and mortality worldwide; for instance, the World Health Organization (WHO) estimates seasonal influenza causes between 250,000 to 500,000 deaths worldwide each year [8] while pneumonia kills more than 4 million people worldwide every year [9]. Worldwide, the morbidity and mortality of influenza and pneumonia have a considerable economic impact in the form of hospital and other health care costs. Each year in the United States approximately 3 million persons acquire pneumonia and, depending on the severity of the influenza season, 15 to 61 million people in the US contract influenza [9]. These numbers contribute to approximately 1.3 million hospitalizations, of which 1.1 million are pneumonia cases [10] and the remainder for influenza [11]. Moreover, pneumonia cases and influenza together cost the American economy 40.2 billion dollars in 2005 [12]. In The Netherlands it has been estimated that influenza accounts for 3713 and 744 days of hospitalization per 100,000 high-risk and low-risk elderly, respectively [13]. Due to the public health burden and the unpredictability of an influenza season, strong pneumonia and influenza surveillance systems are a priority for health authorities.

Mortality monitoring is an important tool for the surveillance of pneumonia and influenza which can aid in the rapid detection and estimates of excess deaths and inform and evaluate the effect of vaccination and control programs. Traditionally, influenza mortality surveillance often uses the category of “pneumonia and influenza” (P-I) on death certificates as an indicator of the severity of an influenza season or to identify trends within a season; however, only a small proportion of these deaths are influenza related. It has been reported that only 8.5–9.8% of all pneumonia and influenza deaths are influenza related [14,15]. The non-influenza-related pneumonia deaths tend to be stable from year to year and fluctuations in this category are largely driven by the prevalence and severity of seasonal influenza. As a result, the P-I category is an important sentinel indicator.

In the US, death certificates are the primary data source for mortality surveillance whose findings are widely used to exemplify epidemics and measure the severity of influenza seasons [16]. Currently, there are three systems to monitor influenza-related mortality; one system in particular, the 122 Cities Mortality Reporting System, provides a rapid assessment of pneumonia and influenza mortality [6]. Each week, this system summarizes the total number of death certificates filed in 122 US cities, as well as the number of deaths due to pneumonia and influenza. However, even these data can be delayed by approximately 2–3 weeks from the times of death. This delay can be attributed to one of the following reasons: 1) timeliness of death registration and 2) reviewing of the death certificates to identify pneumonia and influenza deaths [6,16,17]. The registration and reviewing of death certificates varies by states and, as a result, there is variability in length of time to report a death to CDC. For instance, states with paper-based death registration system typically perform manual reviews of the death certificates which can take up to 3 weeks; however states with electronic death registration systems (EDRS) may perform automatic reviews which can decrease this time significantly.

The current 122 Cities Mortality Reporting System surveillance system also lacks flexibility for expanding the number of conditions and/or the geographic distribution. Moreover, the unavailability of coded death records due to the complexity of the National Center of Health Statistics (NCHS) coding process results in multiple strategies to identify common outbreaks such as pneumonia and influenza deaths, which greatly vary by jurisdiction. To bypass the lengthy NCHS process, a variety of approaches have been attempted that are close to ‘real- time’ but less than optimal. For instance, in Utah keyword searching is used to identify pneumonia and influenza deaths; although this method is fast and easy to implement, it can easily result in the over or under estimation of cases. This can occur by missing cases due to misspelled terms, synonyms, variations, or the selection of strings containing the search term.

Other research groups [18,19] have demonstrated the feasibility of using mortality data for real time surveillance but all used “free text” search for the string “pneumonia”, “flu” or “influenza.” As noted earlier, although this method can provide the semi quantitative measurements for disease surveillance purposes, keyword searches can also result in an array of problems that result from complexities of human language such as causal relationships and synonyms [20]. Therefore, the lack of coded death data that may not be available for months [21] seriously limits the use of death records in automated systems. At this time, there is little published on the automatic assignment of codes to death certificates for automatic case detection.

### Coding death certificates

**Figure 1:**
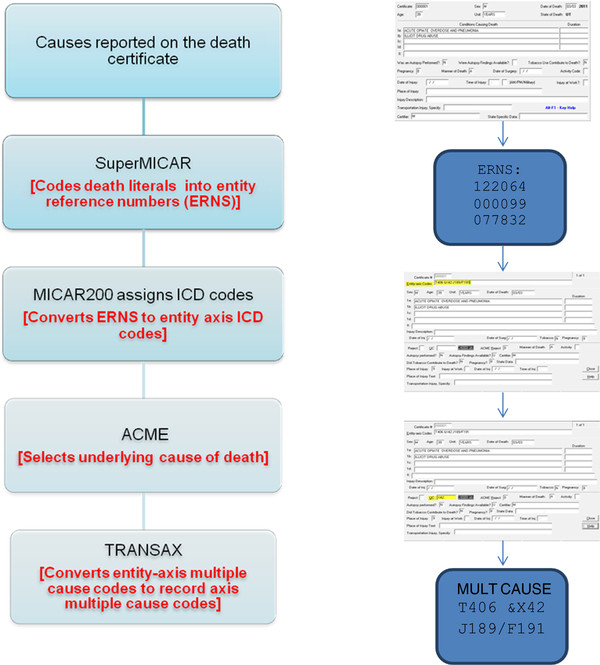
The Mortality Medical Data System (MMDS) Workflow.

Currently the coding of death certificates is a complex process which involves many entities. In the US, where we are focusing this study, the codes on death certificates that are generated by the National Center for Health Statistics (NCHS) **depend on information reported on the death certificate** by the medical examiner, coroner, or another certifier, and there is substantial variation in how certifiers interpret and adhere to cause-of-death definitions [22]. The cause of death literals are coded into International Classification of Diseases Tenth Revision (ICD-10) [23] and the underlying and multiple-cause-of-death codes are selected based on the World Health Organization coding rules. These coding rules have been automated by CDC with the development the Mortality Medical Data System (MMDS) which consists of four programs: Super Mortality Medical Indexing Classification and Retrieval (SuperMICAR) Data Entry; Mortality Medical Indexing Classification and Retrieval (MICAR); Automated Classification of Medical Entities (ACME) and TRANSAX (Translation Axes). SuperMICAR was designed to facilitate the entry of literal text of causes of death in death certificates and convert them into standardized expressions acceptable by MICAR. It contains a dictionary which assigns an entity reference number (ERN) to statements on the death certificate. These ERNs are fed into MICAR200 which transforms the ERNS into ICD-10 codes by using specific mortality coding rules; the rules require look-up files and a dictionary. ACME and TRANSAX then selects the underlying and multiple causes of death respectively. ICD-10 codes from MICAR200 are fed into ACME which assigns the underlying cause of death using decision tables. The decision table contains all possible pairings of diseases for which the first disease can cause the second. In the latest version of the system, ACME is comprised of eight decision tables including three tables of valid and invalid codes, causal relationships (General Principle and Rule 1), and direct sequel (Rule 3), and three other tables needed by modification rules. Figure 1 provides the workflow for the MMDS system.

Of the 2.3 million deaths that occur each year 80–85 percent are automatically coded through SuperMICAR, and the remaining records are then manually coded by nosologists, a medical classification specialist [24]; this is a tedious and lengthy process lasting up to 3 months. Although the automation process has decreased the time required for coding death data to 1–2 weeks, the national vital statistics data is not available for at least two years. Therefore, local health department still manually code records or perform basic process techniques to quickly characterize disease patterns [25].

Records that were processed through Super-MICAR or were manually coded are then processed through the remaining components (MICAR200, ACME and TRANSAX) of MMDS. In 1999, MICAR200 had a throughput rate of 95–97%, while ACME rate was 98 percent. Moreover, based on a reliability study, ACME error rate for selecting the underlying cause is at one-half percent, while TRANSAX, the multiple cause codes had a one-half percent error rate [26]. Due to the high processing rates and low error rates, MMDS is considered by practitioners as the gold standard for the processing and coding of death certificates in the US and other countries (such as Canada, the United Kingdom (UK) and Australia). Therefore, we used the codes produced by this system as the “gold standard” when comparing with the methods developed here.

### Electronic death registration system

In 1997, the US Steering Committee to Reengineer the Death Registration Process (a task force representing federal agencies, the National Center for Health Statistics and the Social Security Administration, and professional organizations representing funeral directors, physicians, medical examiners, coroners, hospitals, medical records professionals, and vital records and statistics officials (NAPHSIS) published the report “*Toward an Electronic Death Registration System in the United States: Report of the Steering Committee to Reengineer the Death Registration Process*.” This report explained the feasibility of developing electronic death registration in the United States [27] and argued that these electronic death records have the potential to be an effective source of information for nation-wide tracking and detecting of disease outbreaks. However, little actions have been taken to implement such recommendations in a comprehensive manner. As of July 2011, electronic death registration systems were operating in 36 states, the District of Colombia, and in development or planning stage in a dozen others [28].

### NLP potential

Information representing the ‘cause of death’ field on the death certificates is free text. One major goal of natural language processing (NLP) is to extract and encode data from free- texts. There have been many research groups developing NLP systems to aid in clinical research, decision support, quality assurance, the automation of encoding free text data and disease surveillance [29-31]. Although, there have been a few NLP applications to the public health domain [32,33], little is known about its capability to automatically code death certificates for outbreak and disease surveillance. Recently, Medical Match Master (MMM) [25], developed by Riedl et al at the University of California Davis, was used to match unstructured cause of death phrases to concepts and semantic types within the Unified Medical Language System (UMLS). The system annotates each death phrase input with two types of information, the Concept Unique Identifier, CUI, and a semantic type both assigned by the UMLS. MMM was able to identify an exact concept identifier (CUI) from the UMLS for over 50% of ‘cause of death’ phrases. Although, the focus of this study was to use NLP techniques to process death certificates, the description of this system reported in the literature did not show how well coded data from an NLP tool along with predefined rules can detect countable cases for a specific disease or condition.

The purpose of our project is to create a pipeline which automatically encodes death certificates using a NLP tool and identify deaths related to pneumonia and influenza which provides daily and/or weekly counts. We compared the new technique developed here with keyword searching and MMDS as exemplars of the easiest possible approach and the current “gold standard”, respectively. The comparison of the techniques was done by calculating recall, precision, F- measure, positive predictive value and agreement (Cohen’s Kappa).

## Methods

### Sample

**Figure 2:**
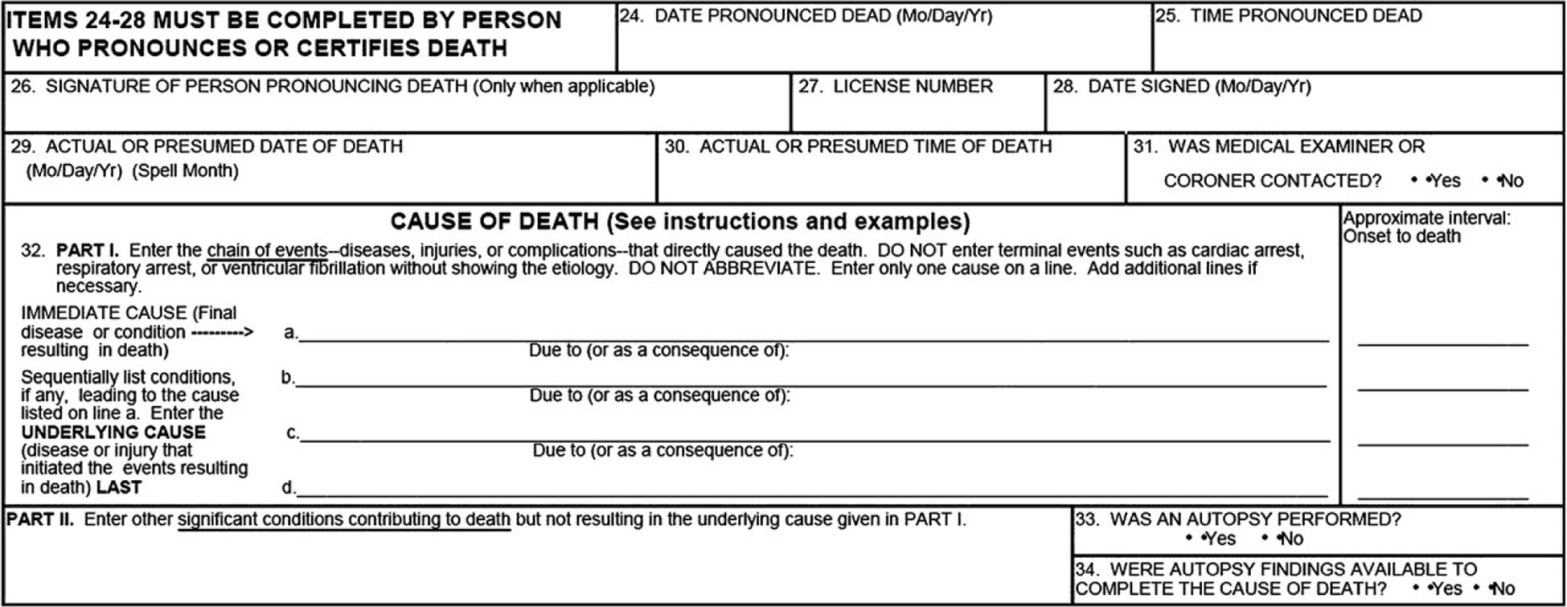
Portion of the US standard certificate of death in which cause-of-death data are entered.

**Figure 3:**
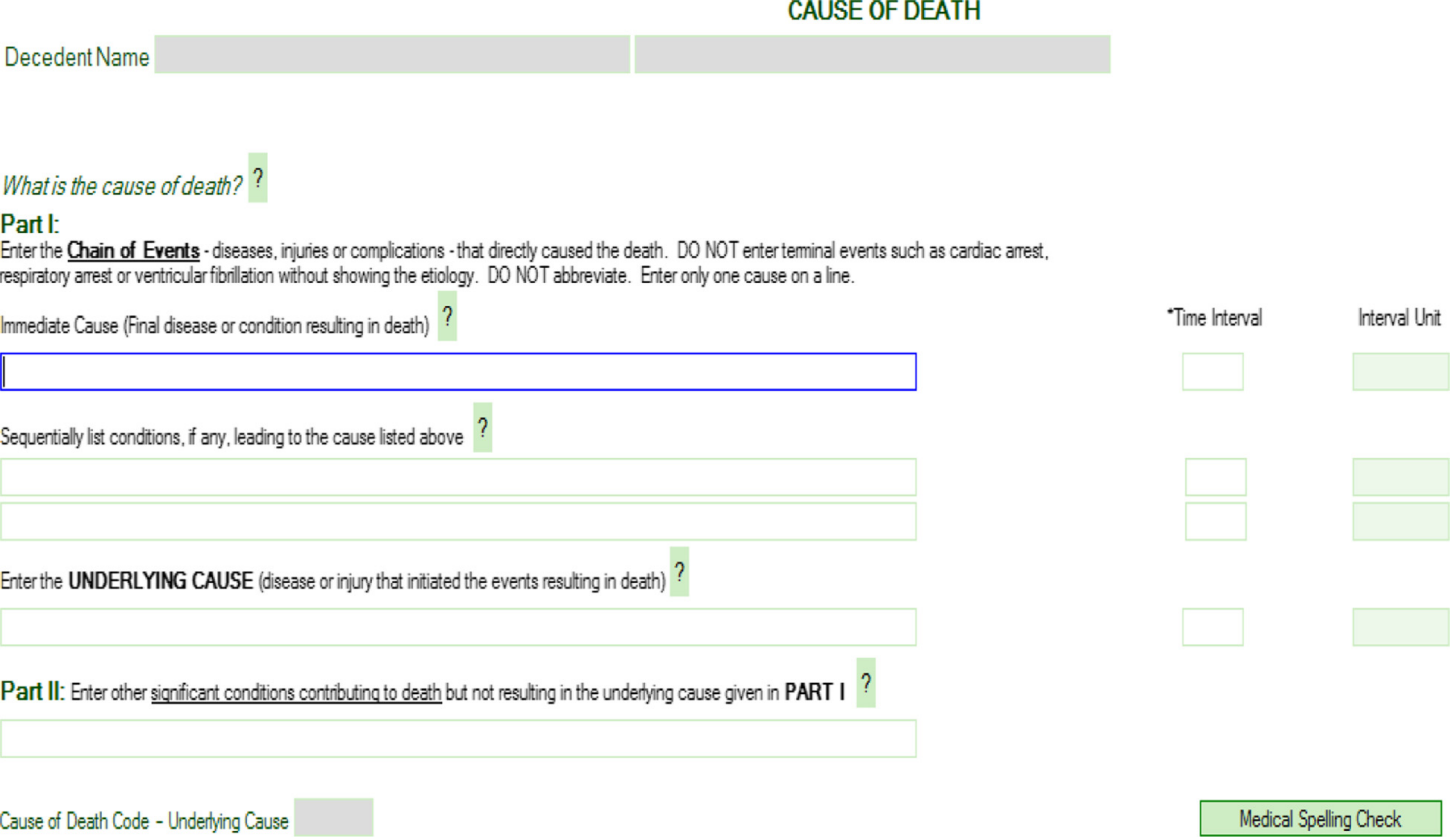
Utah department of health electronic death certificate.

We obtained 14,440 de-identified electronic death records all with multiple-cause-of-death from the Utah Department of Health (UDOH) for the period 1 January 2008 to 31 December 2008. The records included a section describing the disease or condition directly leading to death, and any antecedent causes, co-morbid conditions and other significant contributing conditions. An example of a paper and electronic death certificate are shown in Figures 2 and 3 respectively. All death certificates used in this study have been processed using the Mortality Medical Data System (MMDS) and the record axis codes were received from UDOH.

For our study we randomly selected 6,450 (45%) records. All death records included in the study were previously also coded by NCHS into ICD-10, but this information was not used for our coding, it was only used *as posteriori* to assess to quality of the automatic coding.

### Case definition of pneumonia and influenza deaths

We chose to apply the Centers of Disease Control and Prevention case definition of pneumonia and influenza deaths defined by CDC’s epidemiologist staff through personal communication. Therefore, the operational definition for deaths from influenza includes deaths from all types of influenza with the exception of deaths from HAEMOPHILUS INFLUENZAE infection and deaths from PARAINFLUENZAE VIRUS infection. Pneumonia deaths include deaths from all types of pneumonia including pneumonia due to H. influenza and pneumonia due to parainfluenzae virus. The exceptions include aspiration pneumonia (O74.0, O29, O89.0, J69.- and P24.-)1, pneumonitis (J84.1, J67-J70), and pneumonia due to pneumococcal meningitis (J13, G00.1) 1. Pneumonia and influenza related deaths were defined as one of the diagnoses listed in Table 1 which were reported in any cause of death field. These codes were selected through manual review of the ICD-10 version 2007 manual [23].

**Table 1:** ICD-10 codes relevant to our study

**ICD-10**	**Definition**	**ICD-10**	**Definition**
A01.03	Typhoid fever with pneumonia	B39.0	Pneumonia in acute pulmonary histoplasmosis capsulati
A02.22	Salmonella pneumonia	B39.1	Pneumonia in chronic pulmonary histoplasmosis capsulati
A22.1	Pneumonia in anthrax	B39.2	Pneumonia in pulmonary histoplasmosis capsulati, unspecified
A37.01	Whooping cough in Bordetella pertussis with pneumonia	B44.0	Pneumonia in pulmonary histoplasmosis capsulati, unspecified
A37.11	Whooping cough in Bordetella parapertussis with pneumonia	B44.1	Other pulmonary aspergillosis with pneumonia
A37.81	Whooping cough in other Bordetella species with pneumonia	B44.9	Pneumonia in aspergillosis, unspecified
A37.91	Whooping cough, unspecified species with pneumonia	B58.3	Pneumonia in toxoplasmosis
A42.0	Pneumonia in actinomycosis	B59	Pneumonia in Pneumocystis jiroveci
A42.0	Pneumonia in actinomycosis	B77.81	Ascariasis pneumonia
A43.0	Nocardiosis pneumonia	I00	Rheumatic pneumonia
A48.1	Legionnaires’ disease	J09.-	Influenza due to certadue to identified influenza viruses
A50.04	Early congenital syphilitic pneumonia	J10.-	Influenza in other identified influenza virus
A54.84	Gonococcal pneumonia	J11.-	Influenza in unidentified influenza virus
A69.8	Spirochetal infection NEC with pneumonia	J12.-	Viral pneumonia, not elsewhere classified
A70	Ornithosis	J14.-	Pneumonia in Hemophilus influenzae
B01.2	Varicella pneumonia	J15.-	Bacterial pneumonia, not elsewhere classified
B05.2	Measles pneumonia	J16.-	Pneumonia in other infectious organisms, not elsewhere classified
B06.81	Rubella pneumonia	J17.-	Pneumonia in diseases classified elsewhere
B25.0	Pneumonia in cytomegalovirus disease	J18.-	Pneumonia, unspecified organism
B37.1	Pulmonary candidiasis	J82	Allergic or eosinophilic pneumonia
B38.0	Pneumonia in acute pulmonary Coccidioidomycosis	J95.851	Ventilator associated pneumonia
B38.1	Pneumonia in chronic pulmonary Coccidioidomycosis	Z87.01	Personal history of pneumonia (recurrent)
B38.2	Pneumonia in pulmonary coccidioidomycosis, unspecified		

### Procedures

**Figure 4:**
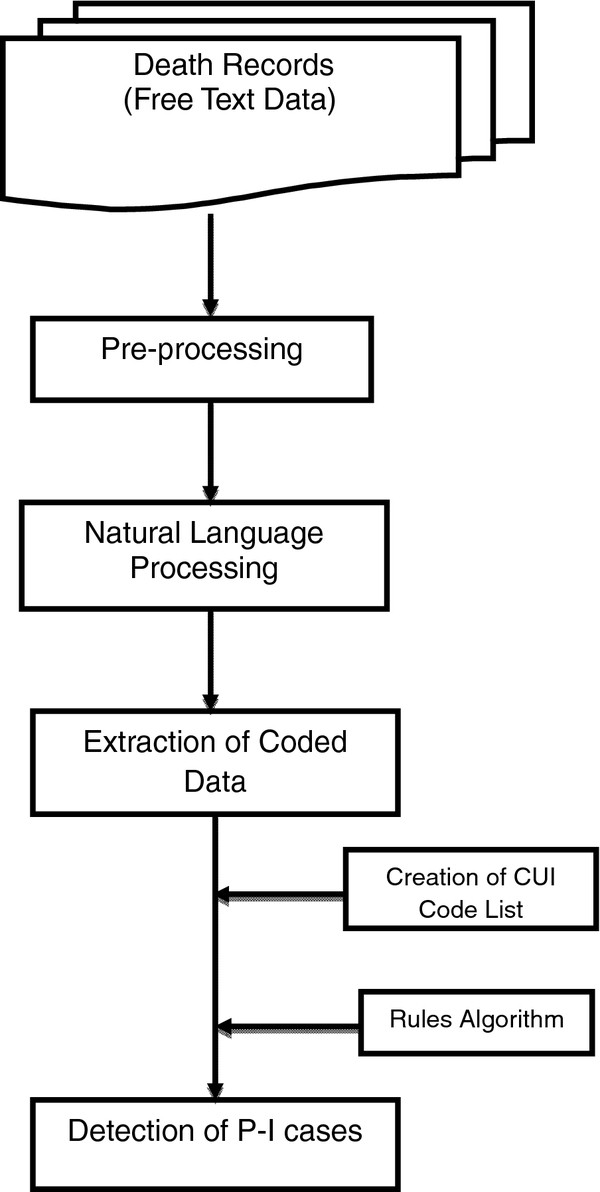
Flow diagram of the death certificates pipeline.

The Death Certificates Pipeline, DCP, was developed to identify pneumonia and influenza cases. The pipeline consisted of two components. The first component of the system was the natural language processor, for which we used MetaMap [34], and the second component was the definitional rules that were applied to the output generated by MetaMap. The study procedures for this pipeline included: preprocessing, NLP, extraction of coded data and the detection of pneumonia and influenza cases (Figure 4).

#### *Step 1: Preprocessing*

Spelling errors are common on death certificates; therefore, the death records were first processed through a spell checker to identify misspellings. Although the UMLS SL has a spell suggestion tool called GSPELL [35-37], we decided not to use it and chose to utilize ASPELL [38]. Our motivation for this decision was based upon an evaluation which showed ASPELL outperforming GSPELL; ASPELL performed better on three areas of performance which were evaluated: (1) whether the correct word was ranked number one; (2) whether the correct word was ranked in the top ten; and (3) whether the correct word was found at all [35]. PERL (http://www.perl.org), a high-level computer programming language that aids in the manipulation and processing of large volume of text data was then used to prepare the cause of death free text for NLP. The preprocessing also involved the removal of non-ASCII characters; this was a required technical step for MetaMap processing.

#### *Step 2: Natural language processing*

MetaMap was used to convert the electronic death records to coded descriptions appropriate for the rule based system. MetaMap [34], developed by the National Library of Medicine (NLM), is useful in identifying biomedical concepts from free-form textual input and maps them into concepts from the Unified Medical Language System (UMLS) Metathesaurus [34,39]. MetaMap works by breaking the inputted text into words or phrases, map them to standard terms, and then match the terms to concepts in the Unified Medical Language System (UMLS) [40]. For each matched phrase, MetaMap classifies it into a semantic type then returns the concept unique identifier (CUI) and the mapping options which are ranked according to the strength of the mapping. Table 2 shows an example of sample death literal and its associated XML output from MetaMap. Text bolded in the output from NLP represent the code and its corresponding phrase.

**Table 2:** Original text and its corresponding metaMap output

**Urinary tract infection, pneumonia**	**Original**	**Snippet of XML output**
	Urinary tract infection,	<Mappings Count="1">
		<Mapping>
		<MappingScore>-1000</MappingScore>
		<Candidates Count="1">
		<Candidate>
		<CandidateScore>-1000</CandidateScore>
		<CandidateCUI>**C0042029**</CandidateCUI>
		**<CandidateMatched>Urinary tract infection</CandidateMatched>**
		<CandidatePreferred>Urinary tract infection</CandidatePreferred>
		<MatchedWords Count="3">
		**<MatchedWord>urinary</MatchedWord>**
		**<MatchedWord>tract</MatchedWord>**
		**<MatchedWord>infection</MatchedWord>**
		</MatchedWords>
		</Candidate>
		</Candidates>
		</Mapping>
		</Mappings>
	pneumonia	<Mappings Count="1">
		<Mapping>
		<MappingScore>-1000</MappingScore>
		<Candidates Count="1">
		<Candidate>
		<CandidateScore>-1000</CandidateScore>
		<CandidateCUI>**C0032285**</CandidateCUI>
		<CandidateMatched>**Pneumonia**</CandidateMatched>
		<CandidatePreferred>Pneumonia</CandidatePreferred>
		**<MatchedWord>pneumonia</MatchedWord>**
		</Candidate>
		</Candidates>
		</Mapping>
		</Mappings>

#### Step 3: Extraction of coded data

The data produced by MetaMap (XML format) was processed through a PERL script to extract the inputted text and its corresponding meta-mapped CUIs. This extracted data was outputted to a text document.

#### Step 4: *Identification of P-I deaths*

The identification of pneumonia and influenza cases involved two steps: 1) identifying CUIs relating to pneumonia and influenza and 2) use of the CUIs to create a rules based algorithm to identify cases. Details of each step are explained in the following paragraphs.

To determine which CUI codes were relevant for identifying pneumonia and influenza deaths it was necessary to create a “CUI code list” that represents all the ICD-10 codes of interest (see Table 1). To create this list, we generated a subset of the UMLS 2010 AB database [41] using the Metamorphosys [40] tool provided by the National Library of Medicine, NLM. The UMLS database includes many vocabularies, therefore, to determine which vocabularies are relevant to our aims we used the procedure used by Riedl *et al*. [25] which included every level 0 source plus SNOMED, a level 9 source. Sources such as National Center for Biotechnology Information (NCBI) taxonomy, which included ICD-10 codes, were also included in our configuration of the subset. For the purpose of our study, we focused on two tables within the UMLS schema. The first table MRCONSO contains a unique row for each lexical variant of a given concept. The second table MRREL contains information about the relationship among concepts. Tables 3 and 4 shows sample rows and columns in the MRCONSO and MRREL tables associated to CUIs related to “pneumonia” [C0032285], “influenza” [C0021400] and “pneumonia and influenza” [C0155870]. Our configuration produced 6,862,110 rows in MCRNOSO and 23, 467,822 rows in MRREL.

**Table 3:** Sample rows and columns from the MRCONSO table

**CUI**	**LUI**	**SUI**	**AUI**	**SAUI**	**SCUI**	**SAB**	**CODE**	**STR**
C0021400	L0021400	S0667823	A17788091	NULL	NULL	ICD10CM	J10.1	Influenza NOS
C0021400	L0016270	S0003527	A2875695	11205017	6142004	SNOMEDCT	6142004	Flu
C0021400	L0018238	S0046068	A2882094	11206016	6142004	SNOMEDCT	6142004	Grippe
C0032285	L3025870	S3482854	A15102770	2.76E + 09	60363000	SNOMEDCT	60363000	Pneumonia (disorder)
C0032285	L0880404	S0991677	A1049957	NULL	NULL	ICD10	J18.9	Pneumonia, unspecified
C0155870	L0182738	S1458440	A1411637	NULL	NULL	ICD10	J10-J18.9	Influenza and pneumonia
C0155870	L0182738	S0247321	A16973811	NULL	NULL	ICD9CM	487	Influenza with pneumonia

**Table 4:** Sample rows and columns from the MRREL Table

**CUI1**	**AUI1**	**STYPE1**	**REL**	**CUI2**	**AUI2**	**STYPE2**	**RELA**	**SAB**	**SL**
C0021400	A0481781	AUI	RB	C0029342	A0318194	AUI	NULL	CSP	CSP
C0021400	A0481781	AUI	RN	C0276357	A1196494	AUI	NULL	CSP	CSP
C0021400	A0412457	AUI	RQ	C0021400	A0247343	AUI	mapped_from	CST	CST
C0032285	A0102675	SDUI	SIB	C0273115	A15577420	SDUI	NULL	MSH	MSH
C0032285	A18169362	CODE	RO	C0485207	A18252567	CODE	has_fragments for_synonyms	LNC	LNC
C0021400	A4386826	CODE	RB	C0348675	A0723374	CODE	mapped_from	ICPC2 ICD 10ENG	ICPC2 ICD 10ENG
C0032285	A1049957	AUI	PAR	C0339951	A0242105	AUI	NULL	ICD10	ICD10
C0155870	A1411637	AUI	CHD	C0339951	A0242105	AUI	NULL	ICD10	ICD10

Three queries were performed on the subset described above to map pneumonia and influenza ICD-10 codes to CUIs and identify related pneumonia and influenza concepts. Each query was then placed in a separate database, all duplicates were removed and a sub-query was run to ensure that only the ICD-10 codes in Table 1 were included in this list. This produced 241 distinct concept identifiers (CUIs) relating to pneumonia or influenza. These codes were used to develop the rules to identify the cases of interest.

**Figure 5:**
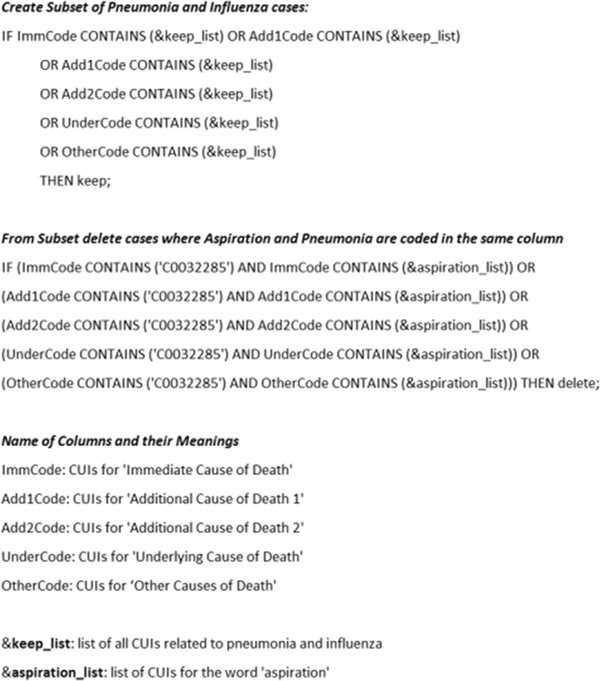
Rules applied to MetaMap’s output to extract pneumonia and influenza cases.

The coded data produced by MetaMap was accessed by rules, aimed at identifying the presence of pneumonia and influenza based on the coded data. The rules for identifying these deaths used the CUI code list described above. The rule looks at each cause of death field (Underlying Cause, Additional Causes, etc.) to flag records with relevant codes. These rules used boolean operators (And, Or, Not) and if-then statements to create a chain of rules (Figure 5).

### Comparison methodologies

The list of cases identified by our automated detection system was compared with those identified by two other methods: a) keyword searching and b) the reference standard: the ICD-10 codes given by the CDC MMDS method. For key-word searching we followed the process utilized by the Utah Department of Health where all the cause of death fields were scanned for the text strings ‘PNEUMONIA’ OR ‘INFLUENZA’. The words ‘ASPIRATION PNEUMONIA’, ‘PNEUMONITIS’, ‘PNEUMOCOCCAL MENINGITIS’, ‘HAEMOPHILUS INFLUENZAE’ and ‘PARAINFLUENZAE VIRUS’ were excluded.

To evaluate the performance of both techniques against the reference standard, we needed to specify what constituted a match. Each death record is associated to a unique number; therefore, we considered a match if the unique identifier was identified by the comparator and also found by the reference standard.

### Statistical analysis

Three standard measures were used to evaluate the performance of one method in relation to the reference standard used in this study: precision (equivalent to positive predictive value; recall (equivalent to sensitivity or true positive rate), and F-measure. Kappa statistics were used to assess agreement and McNemar’s test was used to analyze the significance between the two methods. All calculations were performed in R [42].

To calculate these values, pneumonia and influenza related deaths were examined by comparing the reference standard output vs. the two comparators: DCP and keyword search. For both comparators, the deaths were counted and categorized as TRUE POSITIVES (cases found by the comparator—pneumonia deaths being correctly classified); FALSE POSITIVES (incorrect cases found by the comparator—the number of pneumonia and influenza deaths incorrectly identified by the comparator); FALSE NEGATIVES (correct cases not found by the comparator—the number of pneumonia deaths not identified by the comparator). Precision, recall and F-score were calculated as follows:

Precision = True Positives/(True Positives + False Positives) (1)

Recall = True Positives/(True Positives + False Negatives) (2)

F-measure = 2 *(P R/ P + R) (3)

McNemar’s test was also calculated to evaluate the significance of the difference between the two comparators. To calculate this value a confusion matrix was created where A is the number of times both methods have correct predictions; B is the number of times method 1 has a correct prediction and method 2 has a wrong prediction; C is the number of times method 2 has a correct prediction and method 1 has a wrong prediction; D is the number of times both methods have incorrect predictions.

Ethics approval was not required for this study. Identifying variables that could be used for re-identifying individuals were excluded from the study data.

## Results

### Processing time of the data

The records were processed and analyzed on a server with two Opteron Dual-Core 2.8 GHz processors and 16 GB RAM at the Center of High Performance Computing at the University of Utah. Using keyword searching the CPU processing time to identify pneumonia and influenza cases was 0.21 seconds and the wall time was 0.37 seconds. For the DCP, the total CPU processing time was 881.83 seconds. The NLP portion of the pipeline attributed to 99.4 percent of the processing time (NLP-877 seconds). While the DCP execution time is much longer, still it is well within the “in real time” realm. For instance, it would take 6,364.3 seconds CPU time seconds for DCP to code and flag all the weekly death records of the US (≈ 46,523).

### Statistical analysis

Recall and precision were calculated at a 0.95 confidence intervals; the F-measure was also calculated. The performance of each method is described below.

### Keyword searching

Of the 6,450 records analyzed keyword search identified 473 records as pneumonia and influenza deaths, 21 being identified as false positives. Precision for keyword searching was calculated at 96%. Of the 21 false positives, 6 records correctly mentioned pneumonia in the cause of death text but their corresponding ICD-10 codes failed to provide any code related to pneumonia, while 2 records were flagged because it included the sub-string “pneumonia” in the additional cause of death field. The death literal for these two records were “bacteremia due to Streptococcus pneumonia” and “Streptococcal Pneumoniae Septicemia”, The remaining 13 errors were due to the entry of the death literals; in all cases the negation of ‘aspiration pneumonia’ either due to: 1) ‘pneumonia’ being in a separate cause of death field to ‘aspiration’ or 2) ‘pneumonia’ not being directly followed by ‘aspiration’ in the death text (example “pneumonia due to secondary aspiration”). A total of 20 false negatives were recorded, yielding a recall of 96%. The false negatives could be generalized into two categories: 1) misspellings of pneumonia on the death certificated (n = 8) and 2) appropriate pneumonia or influenza ICD-10 code was coded but the death literals did not mention an appropriate scanned phrase (n = 12). F-measure was also calculated at 96%. A high level of agreement was seen among keyword searching and the reference standard (kappa 0.95).

### Death certificates pipeline

Utilizing the Death Certificates Pipeline (DCP), we identified 481 records as pneumonia and influenza deaths, 9 of which were false positives. The precision for this method was calculated at 98%. Like the keyword searching method, of the 9 false positives, 6 records mentioned pneumonia in the cause of death field but their corresponding ICD-10 codes failed to provide any code related to pneumonia and the remaining errors were due to the reporting of aspiration pneumonia on the death certificate. This method had only 1 false negative for the death literal stating “recurrent aspiration with pneumonia”, thus yielding a recall at 99.8%, being less than keyword searching. F-measure was calculated at 99%. The level of agreement between the pipeline and the gold standard was almost perfect with a Cohen’s kappa of 0.988.

The precision and recall scores that are reported above suggest that the DCP is a better method for identifying pneumonia and influenza deaths than keyword-searching. Therefore, we investigated if this observation is supported by statistical analysis. Performing a Fisher’s exact test at *α* = 0.05, significant difference was seen for both recall (*p* = 1.742e-05) and precision (*p* = 0.026). The McNemar’s test result also showed DCP to be a better method with a *p*-value = 2.152e-05.

### Analysis of failures

For the 472 pneumonia and influenza cases found by the reference standard, DCP correctly identified 471 cases, missed one case and incorrectly flagged nine cases. Most failures were due to discrepancies between the death literal and its respective ICD-10 code. For the only case which the pipeline did not match, the phrase ‘recurrent aspiration with pneumonia’ was present in the death literal. MetaMap coded this literal as aspiration pneumonia which was excluded from the CUI code list, but its respective ICD-10 included J189. For the 9 additional cases which were not present in the reference standard, we noticed two categories of errors: 1)cases where the string ‘pneumonia’ is present in the death literal but not coded into ICD-10 and 2) the reporting of aspiration pneumonia on the death certificate. The first category of errors was not due to MetaMap or the rule algorithm, but perhaps due to the coding process. As described earlier, MMDS produces entity axis and record axis codes. The entity axis codes would be a more appropriate reference standard for they provide the ICD 10 codes for the conditions or events reported as listed by the death certifier and maintains the order as written on the death certificate [43]; but as noted earlier only the record axis codes were made available for this study. The algorithm used to produce record axis codes from the entity axis data removes duplicate codes and contradictory diagnoses within the entity axis data to produce the more standardized record axis [44]. For example, if a medical examiner reports pneumonia with chronic obstructive pulmonary disease both conditions will be shown in entity axis code data. However, in record axis code data, they will be replaced with a single condition: Chronic obstructive pulmonary disease with acute lower respiratory infection (J44.0). We were unable to verify that codes related to pneumonia were present in the entity axis codes for the six cases; therefore, we can only speculate the reason for this failure.

The second category of errors was due to the reporting of aspiration pneumonia on the death certificate. In cases where the string “aspiration” and pneumonia” were not reported in the same text field MetaMap processed the string separately thus yielding two codes: one for aspiration and the other pneumonia, instead of one code for “aspiration pneumonia” [C0032290]. In an initial review of MetaMap we found MetaMap had difficulties processing the phrase “pneumonia secondary to acute aspiration”, therefore, our rule detection algorithm excluded cases where the code for pneumonia and aspiration were present in the same text field.

## Discussion

To our knowledge, this is the first published report on using a natural language processing tool and the UMLS to identify pneumonia and influenza deaths from death certificates. We found that automated coding and identification of pneumonia and influenza deaths is possible and computationally efficient. The Death Certificates Pipeline developed here was statistically different to keyword searching and has higher recall and precision when compared to the current semi-automatic methods in use by the CDC. A good recall is required to help capture the ‘true’ P-I deaths and a good precision is needed to avoidoverestimating the number of P-I deaths. This study also indicated that keyword searching underestimated pneumonia and influenza deaths in Utah. The simple keyword search method not only decreased recall and precision but also reduced the level of agreement. When reporting counts for surveillance purposes it’s best to be as accurate as possible; however, there’s a trade-off between recall and precision. For disease surveillance, increased precision enables public health officials to more accurately focus resources for control and prevention, therefore, although both methods had good precision the pipeline developed would be more advantageous to utilize.

MetaMap did an excellent job at extracting cause of deaths from free-form text which is consistent with the results of Reid et Al [25]. Most of the concepts were present in the UMLS which attributed good recall. Both recall and precision depended on the comprehensiveness of the CUI code list. The performance of this system is determined largely by the coverage of terms and sources in the UMLS. Both keyword searching and the system’s weakest point is its lack of precision. Most of the concepts the system did not identify had either the aspiration text in another field or pneumonia was mentioned in the cause of death text but not coded (9 cases fit these criteria). The sample size was sufficient to show difference between the two methods. It is important to note that utilizing trained nosologists, who would manually code the death certificates, would have developed an absolute gold standard which may or may not be a better reference standard than ICD-10 codes. However, our motivation for utilizing ICD codes was influenced due to the fact that the use of ICD codes to identify all-cause pneumonia has been examined and has showed to be a valid tool for the identification of these cases [45,46].

In terms of timing, while keyword searching is faster than the DCP, our method is also sub 1/10 second range, which implies that it is possible to process the daily Utah deaths (~40) in approximately 5.47 seconds and all deaths in the US (~ 6646) in approximately 909.17 seconds using current hardware. This timing would be much faster than the minimum of two weeks to receive the coded data from the current CDC process. Moreover, these timings make it apparent that this system can be integrated in a real time surveillance system without introducing any additional bottlenecks.

There are several potential limitations with this analysis. First, the generalizability of the findings is limited because the death records were only from one institution. Although death certificates have a standardized format, the death registration process and the reviewing of death records differ by institutions. UDOH utilizes keyword searching to identify pneumonia and influenza cases, other institutions may use more accurate (manual review) or less accurate methods for finding cases. Second, a separate evaluation of the NLP component of the DCP was not performed. Further research is needed to examine the use of NLP on electronic death records across institutions and countries which may have different documentation procedures.

### Conclusions and future work

This study shows that it is feasible to achieve high levels of accuracy when using NLP tools to identify cases of pneumonia and influenza cases from electronic death records while still providing a system that can be used for real time coding of death certificates. Identification of concept identifiers related to the CDC’s case definition of pneumonia and influenza was very important in producing a highly accurate rule for the identification of these cases. Future work will aim to improve the preprocessing phase of the pipeline by providing the inclusion of the spellchecker used by the CDC’s Mortality Medical Data System. Future work will also involve evaluating the flexibility (e.g. identification of different diseases) of the system to deploy the pipeline tool, along with other public health related analytical tools, as a grid service to provide to real time public health surveillance tool that uses data and services under the control of different administrative domains.

We have shown that it is feasible to automate the coding of electronic death records for real-time surveillance of deaths of public health concern. The performance of the Pipeline outperformed the performance of current methods, keyword searching, in the identification of pneumonia and influenza related deaths from death certificates. Therefore, the Pipeline has the potential to aid in the encoding of death certificates and is flexible to identify deaths due to other conditions of interest as the need arises.

## Competing interests

The authors declare that they have no competing interests.

## Authors’ contributions

All the authors contributed equally to this research. All authors read and approved the final manuscript.

## Pre-publication history

The pre-publication history for this paper can be accessed here:

http://www.biomedcentral.com/1472-6947/12/37/prepub
